# Exploring the Complexity of the Interaction between *T. rubrum* and *S. aureus*/*S. epidermidis* in the Formation of Polymicrobial Biofilms

**DOI:** 10.3390/microorganisms12010191

**Published:** 2024-01-18

**Authors:** Jenyffie A. Belizario, Níura M. Bila, Carolina O. Vaso, Caroline B. Costa-Orlandi, Matheus B. Mendonça, Ana M. Fusco-Almeida, Regina H. Pires, Maria José S. Mendes-Giannini

**Affiliations:** 1 Department of Clinical Analysis, School of Pharmaceutical Sciences, São Paulo State University (U.N.E.S.P.), São Paulo 14800-903, Brazil; jee.abelizario@gmail.com (J.A.B.); niura.madalena.bila@gmail.com (N.M.B.); carolovaso@hotmail.com (C.O.V.); carolbarceloscosta@gmail.com (C.B.C.-O.); matheus.bordoy@unesp.br (M.B.M.); ana.marisa@unesp.br (A.M.F.-A.); 2 Department of Para-Clinic, School of Veterinary, Eduardo Mondlane University (UEM), Maputo 257, Mozambique; 3 Postgraduate Program in Health Promotion, University of Franca, São Paulo 14404-600, Brazil; regina.pires@unifran.edu.br

**Keywords:** dual-species biofilms, biofilms, dermatophytes, *Trichophyton rubrum*, *Staphylococcus aureus*, *Staphylococcus epidermidis*

## Abstract

Dermatophytes associated with bacteria can lead to severe, difficult-to-treat infections and contribute to chronic infections. *Trichophyton rubrum*, *Staphylococcus aureus*, and *Staphylococcus epidermidis* can form biofilms influenced by nutrient availability. This study investigated biofilm formation by these species by utilizing diverse culture media and different time points. These biofilms were studied through scanning electron microscopy (SEM), confocal laser scanning microscopy (CLSM), biomass, metabolic activity, and colony-forming units (CFUs). The results revealed that mixed biofilms exhibited high biomass and metabolic activity when cultivated in the brain heart infusion (BHI) medium. Both bacterial species formed mature biofilms with *T. rubrum* within 72 h, irrespective of media. The timing of bacterial inoculation was pivotal in influencing biomass and metabolic activity. *T. rubrum*’s development within mixed biofilms depended on bacterial addition timing, while pre-adhesion influenced fungal growth. Bacterial communities prevailed initially, while fungi dominated later in the mixed biofilms. CLSM revealed 363 μm thick *T. rubrum* biofilms with septate, well-developed hyphae; *S. aureus* (177 μm) and *S. epidermidis* (178 μm) biofilms showed primarily cocci. Mixed biofilms matched *T. rubrum*’s thickness when associated with *S. epidermidis* (369 μm), with few hyphae initially. Understanding *T. rubrum* and Staphylococcal interactions in biofilms advances antimicrobial resistance and disease progression knowledge.

## 1. Introduction

Dermatomycoses, infections that affect the skin and/or appendages, are widespread dermatological diseases, impacting over one billion individuals regardless of gender, race, color, or age [1,2]. Among the main agents causing the diseases are dermatophyte fungi such as *Trichophyton rubrum* and *Candida* yeasts, which are frequently implicated. These diseases are commonly found in regions with tropical and subtropical climates, where favorable temperature and humidity conditions promote their growth. Untreated or inadequately managed dermatophytosis can lead to highly infectious and recurrent clinical manifestations, adversely affecting patients’ quality of life, and may result in concurrent lesions and secondary bacterial infections [3,4,5].

Interactions between fungal and bacterial species are prevalent in the environment and can occur during the colonization of human surfaces, potentially influencing microbial pathogenesis, mainly when biofilms are formed [6,7]. Despite the skin being colonized by various bacteria, only three bacterial genera—*Staphylococcus*, *Propionibacterium*, and *Corynebacterium*—are clinically relevant [8]. *Staphylococcus* stands out due to its global prevalence, especially compared to its species, *S. epidermidis* and *S. aureus*. The former is frequently found on the skin and mucous membranes, especially in humid areas [9,10]. Despite traditionally being considered a non-pathogenic or less aggressive commensal microorganism, recent reports have identified *S. epidermidis* as an opportunistic pathogen associated with *T. rubrum* in cases of *tinea pedis* [5]. This aspect suggests that bacterial strains might play a role in developing these manifestations, especially in interdigital areas, intensifying the disease’s aggressiveness and leading to high inflammation due to skin erosion and maceration caused by bacterial superinfection [5,11,12]. *Staphylococcus aureus*, another species within the genus, is known to cause a variety of acute and chronic infections in different parts of the body, including secondary infections in existing wounds, and is linked to opportunistic infections in immunocompromised and hospitalized patients [13,14]. While the in vivo association between *T. rubrum* and *S. aureus* has not been reported to date, complex cases of *T. rubrum* infection have been documented in immunocompromised patients [15,16,17].

In a natural microbiota environment, the contributions of various microorganisms to the dynamics of infections caused by *T. rubrum* can be complex. The association between dermatophytes and bacteria can intensify superficial fungal infections’ aggressiveness and inflammatory nature [5]. Recent research by our group revealed antagonistic interactions between some prevalent species in dermatophytosis, such as *C. albicans*/*C. parapsilosis* and *T. rubrum*, suggesting that the presence of the dermatophyte prevents the filamentation of *C. albicans* and the development of *C. parapsilosis*, which are essential factors in fungal virulence [18].

However, the significance of *T. rubrum* biofilms in conjunction with skin microbiota bacteria, especially *Staphylococcus* species, remains largely unexplored. The in vitro polymicrobial biofilms pose notable challenges due to their intricate nature, diverse culture media composition, and resistance to antimicrobial agents. In addition, they constitute a better predictive alternative in polymicrobial infections in patients and the possibility of studying factors that influence the establishment, maintenance, and eradication of these infections. Therefore, this study aims to evaluate the ability of *T. rubrum* to form biofilms in vitro in association with *S. aureus* and *S. epidermidis* under different growth conditions and nutrient sources.

## 2. Materials and Methods

### 2.1. Microrganisms

The biofilm-forming strains of *T. rubrum* INCQS 40051 (ATCC 28189) and the strains *S. epidermidis* INCQS 00016 (ATCC 12228) and *S. aureus* INCQS 00015 (ATCC 25923) were used [19,20,21]. The dermatophyte was cultivated in malt extract agar (comprising 2% malt extract (Kasvi), 2% peptone from animal tissue 2% (Sigma-Aldrich, Milano, Italy), 2% glucose (Dinâmica Química Contemporânea LTDA, Indaiatuba, São Paulo, Brazil), and 2% agar (Kasvi- São José dos Pinhais, Paraná, Brazil) and maintained at 25 °C for 7 days or until sporulation [14,18,19]. Conversely, the bacteria were seeded on Typtone Soya Agar (TSA) (Kasvi-Sao Jose dos Pinhais, Paraná, Brazil) and incubated at 37 °C for 12 h.

### 2.2. In Vitro Monospecies Biofilm Formation

Three different culture media were employed in the study. These included: (i) Roswell Park Memorial Institute (RPMI) 1640 (Gibco^®^, Thermo Fisher Scientific, Waltham, MA, USA) supplemented with L-glutamine and devoid of sodium bicarbonate, along with 2% glucose (Dinâmica Quimica Contemporânea LTDA–Indaiatuba, São Paulo, Brazil) and buffered with morpholino propanesulfonic acid (MOPS-Sigma-Aldrich, Milano, Italy), (ii) brain heart infusion broth (BHI) (Kasvi-Sao Jose dos Pinhais, Parana, Brazil), and (iii) Mueller–Hinton broth (MHB) (Kasvi-Sao Jose dos Pinhais, Parana, Brazil). RPMI-1640 was selected due to its established usage in fungal biofilm studies [19,22,23,24]. BHI was chosen for its suitability in exploring mixed biofilms involving yeasts and bacteria [25,26,27]. Mueller–Hinton broth was used due to its use for cultivating bacterial biofilms [28,29].

Biofilms of *T. rubrum* were cultivated following previously published protocols [19,20,21,22,23,24,25,26,27,28,29,30]. Conidia were adjusted to a final concentration of 1 × 10^6^ cells/mL, and a 200 µL inoculum was added to each well of a 96-well microplate. The plates were then statically incubated at 37 °C for 4 h for the pre-adhesion stage. The supernatant was removed, and non-adherent cells were washed with phosphate-buffered saline (PBS). Subsequently, 200 µL of RPMI-1640, BHI, and MHB medium were added to the respective wells, and the plates were incubated at 37 °C for up to 72 h without agitation to allow biofilm development. Biofilms of *S. epidermidis* and *S. aureus* were formed following the procedure outlined by Stepanović et al. (2007) [31] with minor adjustments. The strains were aerobically cultivated on TSA agar for 12 h at 37 °C. After incubation, the inoculum was prepared in the corresponding culture media (RPMI-1640, BHI, and MHB) by adjusting the turbidity to McFarland’s 0.5 standard, approximately ~1 × 10^8^ cells/mL. A 200 µL aliquot was seeded in the wells of 96-well microplates and incubated at 37 °C for up to 72 h, following the same maturation time as the *T. rubrum* biofilm [18,19].

### 2.3. In Vitro Formation of Mixed Biofilms

Fungal and bacterial suspensions were prepared separately to form mixed biofilms, following the procedures described in the previous section. The bacterial inoculum of either *S. aureus* or *S. epidermidis* was then combined with the fungal inoculum at different stages of biofilm formation: (i) 0 h—fungal and bacterial inocula were co-cultivated simultaneously; (ii) 4 h—the bacterial inoculum was added after the adhesion phase of *T. rubrum*; (iii) 24 h—the bacterial inoculum was added after 24 h of *T. rubrum* biofilm formation; and (iv) 48 h—the bacterial inoculum was added after 48 h of *T. rubrum* biofilm formation. All mixed biofilms were formed using RPMI-1640, BHI, and MHB media and were incubated for 72 h at 37 °C.

### 2.4. Biofilm Biomass Quantification

The crystal violet (CV) assay was employed to quantify monospecies and mixed biofilms, which involved the following steps: methanol fixation, 0.1% crystal violet staining, and 33% acetic acid decolorization. The optical densities were measured using a microplate reader (Biotek Epoch 2), and the consequent absorbance values were directly proportional to the amount of biofilm biomass [18,32,33,34].

### 2.5. Biofilm Metabolic Activity Quantification

The metabolic activity of both monospecies and mixed biofilms was assessed using the 2,3-bis(2-methoxy-4-nitro-5-sulfophenyl)-5-[carbonyl(phenylamino)]-2H-tetrazolium (XTT) hydroxide reduction test. XTT is a redox dye that is reduced to formazan salt (orange) in metabolically active cells [28,29,30,31,32]. In eukaryotes, XTT reduction occurs through mitochondrial activity, while in prokaryotes, it occurs through an active electron transport system [35,36]. Solutions of XTT (1 mg of XTT/mL in PBS) and menadione (1 mM in PBS) were employed in the assay. After forming biofilms in culture media and incubating times in 96-well plates, the supernatants were carefully removed, and the biofilms were washed with sterile PBS. Next, 50 µL of XTT solution and 4 µL of menadione solution were added to the biofilm wells. The plates were then incubated at 37 °C and shielded from light, for three hours. Subsequently, cell viability was measured using a spectrophotometer (EpochTM 2, Biotek Instruments, Santa Clara, CA, USA) at 490 nm [37].

### 2.6. Biofilm Quantification by the Total Plate Count Method

After the formation of biofilms in 96-well microplates, the supernatant was removed, and 200 μL of sterile PBS was added to each well. The biofilms were gently scraped from the bottom of the wells using sterile tips to dislodge and collect the biofilm cells. The contents were then transferred to microtubes and vortexed to disperse the biofilm cells. Serial dilutions were prepared in PBS, and 10 μL aliquots were inoculated onto Mycosel agar (Difco; BD Biosciences) (for the isolation of *T. rubrum*) and Mannitol salt agar (Kasvi) (for the isolation of *S. aureus* and *S. epidermidis*). The Mycosel agar plates were incubated at 28 °C for up to 14 days, while the Mannitol salt agar plates were incubated at 37 °C for up to 48 h. After the appropriate incubation periods, the colonies obtained were converted into logarithmic colony-forming units per milliliter (CFUs/mL) [18,19,20,21,22,23,24,25,26,27,28,29,30,31,32,33,34,35,36,37,38].

### 2.7. Analysis of Biofilms with Scanning Electron Microscopy (SEM)

The biofilms formed at the bottom of the 24-well plates was washed with sterile PBS, fixed with 800 µL of a 2.5% glutaraldehyde solution (Sigma-Aldrich, Milano, Italy), and incubated under refrigeration at 4 °C for 1 h. Subsequently, the plates were washed, and the samples were dehydrated with increasing concentrations of ethyl alcohol from 50% to 100% at room temperature [18,19,39]. Then, the bottom of the plate containing the samples was cut using a scalpel, mounted on aluminum with silver cylinders (stubs), and placed in a high vacuum metallizer (Denton Vacuum Desk V, Jeol, Moorestown, NJ, USA) for gold coating. Biofilm topographies were evaluated using a scanning electron microscope (Jeol JSM-6610LV, Moorestown, NJ, USA). The architectures of the mixed biofilms established in the four experimental situations were analyzed and compared with the monospecies biofilms after 72 h of incubation.

### 2.8. Analysis of Biofilms with Confocal Laser Scanning Microscopy (CLSM)

To analyze the biofilms, Calcofluor White (Sigma-Aldrich Milano, Italy) and SYTO™ 9 485/498 (Thermo Fisher Scientific, Waltham, MA, USA) fluorochrome solutions were prepared following the manufacturers’ recommendations. Calcofluor White is a fluorophore with high sensitivity and specificity to fungal cell wall chitin, resulting in blue staining of the cell walls [40]. On the other hand, SYTO™ 9 binds to the deoxyribonucleic acid (DNA) of viable cells and can diffuse through intact bacterial membranes, providing indirect information on the total biomass of the biofilm, which appears green when visualized [41]. Following previously published protocols, monospecies and mixed biofilms were formed in 24-well plates with sterile glass coverslips [30,31,32,33,34,35,36,37,38,39,40,41,42]. After the 72 h incubation period at 37 °C, the supernatant was carefully removed, and the plates were centrifuged and washed with PBS to eliminate non-adherent cells. Next, 1 mL of 4% paraformaldehyde was added, and the plates were incubated at 4 °C overnight. After this step, the plates were centrifuged again and washed with PBS. For staining, 200 μL of Calcofluor White solution at 100 mg/L was added to the wells, and the plates were incubated for 30 min at 37 °C and protected from light. Excess dye was removed by washing the plates with PBS. Subsequently, 200 μL of SYTO 9 solution (diluted 1:1000) was added to the wells and incubated for 1 h at room temperature, and they were protected from light. The wells were rewashed with PBS, and the coverslips were mounted on microscopy slides under 4 μL of Fluoromount-G (Sigma-Aldrich, Milano, Italy) for observation. The biofilm samples were observed using a confocal fluorescence microscope (Carl Zeiss LSM 800 with Airyscan). The acquired images were then analyzed using Zen Blue 3.2 software (Carl Zeiss, Jena, Germany), and ImageJ-win64.ex software was used for further processing and in-depth analysis of the obtained images.

### 2.9. Statistical Analysis

All experiments were conducted in triplicate, and three independent experiments were performed. We employed the one-way ANOVA test for the parametric data obtained and the Bonferroni test as a post-test. Statistical significance was defined as *p*-values less than 0.05. GraphPad Prism 5.0 Software (GraphPad Software Inc., San Diego, CA, USA) was used for data analysis.

## 3. Results

### 3.1. Biomass Quantification by the Crystal Violet Assay

**Figure 1:**
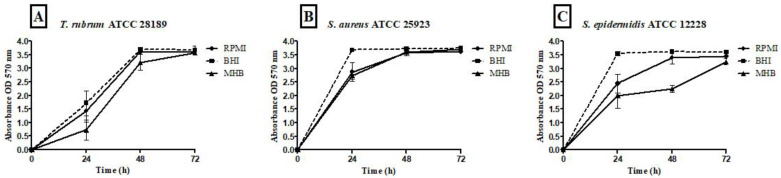
Quantification of biomass performed using the crystal violet (CV) assay and measuring the absorbance at 570 nm from biofilms of *T. rubrum* ATCC 28189, *S. epidermidis* ATCC 12228, and *S. aureus* ATCC 25923. Biofilms formed by *T. rubrum* (**A**) showed lower biomasses at 24 and 48 h in the MHB medium when compared to the RPMI 1640 (*p* < 0.01 and 0.001, respectively) and BHI (*p* < 0.001); *S. aureus* ATCC 25923 (**B**) biofilms also exhibited greater growth in BHI medium compared to RPMI 1640 (*p* < 0.001 and *p* < 0.01) and MHB (*p* < 0.001 and *p* < 0.5) in 24 and 48 h, respectively. In 72 h, statistical differences were observed between RPMI vs. BHI (*p* < 0.001) and RPMI vs. MHB (*p* < 0.01). For *S. epidermidis* ATCC 12228 biofilms (**C**), the BHI stimulated greater biomass compared to RPMI 1640 (*p* < 0.001; *p* < 0.5, *p* < 0.001) and MHB (*p* < 0.001) in 24, 48, and 72 h, respectively. Statistical differences were also observed between RPMI vs. MHB (*p* < 0.01; *p* < 0.001) in 48 and 72 h.

**Figure 2:**
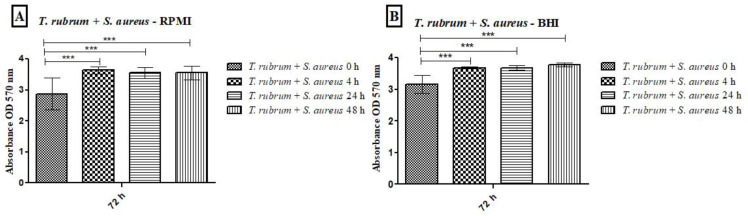

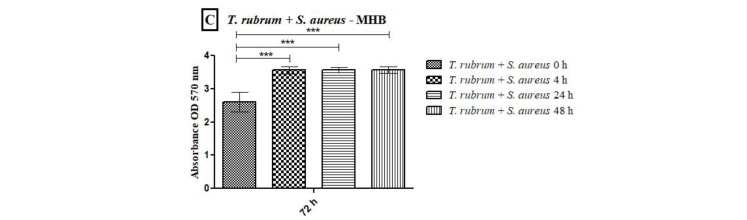
Biomass quantification of biofilms formed by *T. rubrum* + *S. aureus* performed using the crystal violet assay (CV) in RPMI-1640 (**A**), BHI (**B**), and MHB (**C**) media. The times (0 h, 4 h, 24 h, and 48 h) represent the periods during which the bacteria were added together with *T. rubrum*, with a total incubation time of 72 h. Statistical significance (*** *p* < 0.001) is indicated.

**Figure 3:**
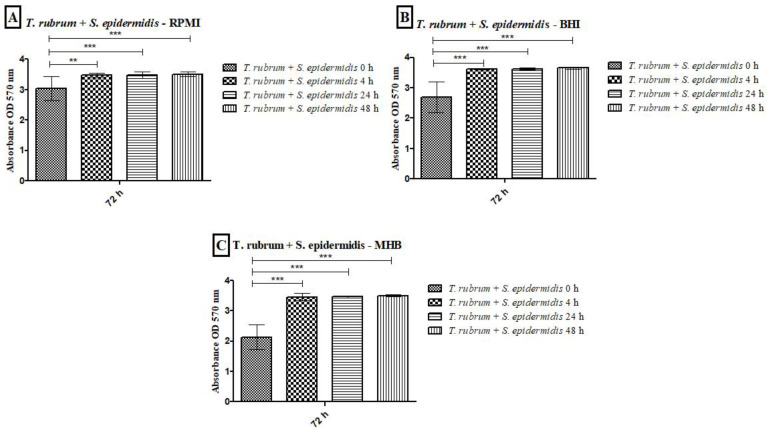
Biomass quantification of biofilms formed by *T. rubrum* + *S. epidermidis* was performed using the crystal violet assay (CV) in RPMI-1640 (**A**), BHI (**B**), and MHB (**C**) media. The times (0 h, 4 h, 24 h, and 48 h) represent the periods during which the bacteria were added together with *T. rubrum*, with a total incubation time of 72 h. Statistical significance levels (** *p* < 0.01 and *** *p* < 0.001) are indicated.

All tested media supported biofilm formation, with varying biomass production (Figure 1). *T. rubrum* biofilm (Figure 1A) exhibited exponential growth up to 48 h, with higher biofilm mass in RPMI-1640 and BHI compared to MHB (*p* < 0.001), followed by a plateau at 72 h. *S. aureus* biofilms (Figure 1B) showed significant biomass in the first 24 h in all media, with BHI promoting the highest growth (*p* < 0.001). RPMI-1640 yielded the lowest biomass (*p* < 0.01). *S. epidermidis* biofilms (Figure 1C) in BHI showed the highest biomass at 24 h (*p* < 0.001) and remained stable until 72 h, while MHB had the lowest biomass (*p* < 0.001). In general, the BHI medium favored the growth of biomass of *Staphylococcus* species biofilms; on the other hand, *T. rubrum* biofilms were favored by BHI and RPMI 1640. For mixed *T. rubrum* + *S. aureus* biofilms (Figure 2A–C), simultaneous placement of microorganisms (0 h) led to significantly lower biomass compared to 4 h, 24 h, and 48 h (*p* < 0.001). Similar trends were observed for mixed *T. rubrum* + *S*. *epidermidis* biofilms (Figure 3A–C) (*p* < 0.01). The differences were not considered significant when bacteria were added after 4 h, 24 h, and 48 h of fungal biofilm development (Figure 2 and Figure 3).

### 3.2. Biofilm Metabolic Activity Quantification

**Figure 4:**
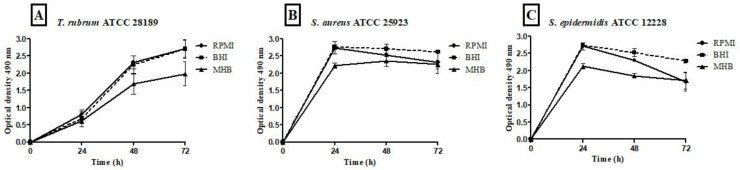
Quantification of metabolic activity by the XTT reduction assay at 490 nm of biofilms of *T. rubrum* ATCC 28189, *S. aureus* ATCC 25923, and *S. epidermidis* ATCC 12228 grown in the media BHI, MHB, and RPMI-640. *T. rubrum* biofilms showed exponential growth up to 72 h; however, the MHB medium showed less metabolic activity than BHI and RPMI-1640 (*p* < 0.001) at 48 and 72 h (**A**). For *S. aureus* ATCC 25923 (**B**) and *S. epidermidis* ATCC 12228 (**C**), metabolic activity growth peaked in the first 24 h in all media, followed by maintenance/a slight decrease until 72 h. The MHB medium stimulated lower metabolic activity than BHI and RPMI (*p* < 0.001) at 24 h. At 48 h for *S. aureus* ATCC 25923 (**B**), the BHI medium showed significance concerning MHB (*p* < 0.001), and at the 72 h time point, the BHI medium showed a significant difference between the MHB and RPMI (*p* < 0.01). For biofilms formed by *S. epidermidis* ATCC 12228 (**C**), there were statistically significant differences in the MHB medium regarding BHI and RPMI (*p* < 0.001) and BHI vs. RPMI (*p* < 0.001) at 48 h. At 72 h, BHI showed significant differences compared to MHB and RPMI (*p* < 0.001).

The metabolic activity kinetics of monospecies biofilms formed by *T. rubrum*, *S. aureus*, and *S. epidermidis* with different culture media are illustrated in Figure 4A–C. The metabolic activity quantification of *T. rubrum* biofilms showed a substantial increase up to 72 h in all tested media (Figure 4A); however, comparing the three media, MHB communities exhibited significantly lower metabolic activity than BHI and RPMI-1640 (*p* < 0.001) at 48 h and 72 h. For *S. aureus* (Figure 4B) and *S. epidermidis* biofilms (Figure 4C), BHI and RPMI-1640 promoted higher metabolic activity at 24 h compared to MHB (*p* < 0.001). The BHI and MHB media maintained constant metabolic activities from 24 h for up to 72 h. The bacterial communities formed in the RPMI medium showed a slight decrease in metabolic activity.

**Figure 5:**
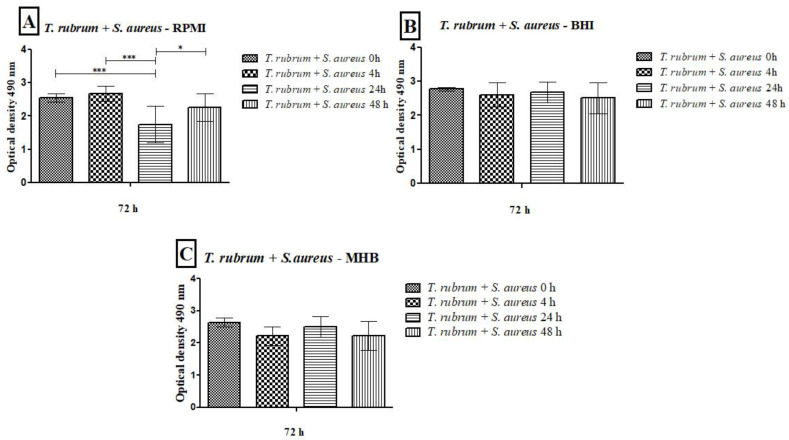
Quantification of metabolic activity by the XTT reduction assay of biofilms formed by *T. rubrum* + *S. aureus* in RPMI-1640 (**A**), BHI (**B**), and MHB (**C**). The times (0 h, 4 h, 24 h, and 48 h) represent the periods during which the bacteria were added together with *T. rubrum*, with a total incubation time of 72 h. Statistical significance levels (* *p* < 0.05 and *** *p* < 0.001) are indicated.

**Figure 6:**
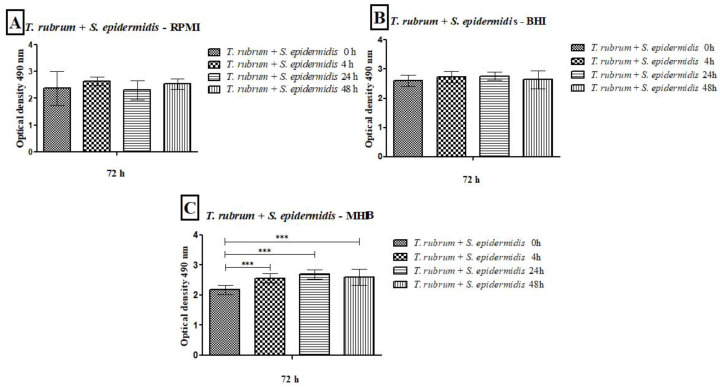
Quantification of metabolic activity by the XTT reduction assay of biofilms formed by *T. rubrum* + *S. epidermidis* in RPMI-1640 (**A**), BHI (**B**), and MHB (**C**). The times (0 h, 4 h, 24 h, and 48 h) represent the periods during which the bacteria were added with *T. rubrum*, with a total incubation time of 72 h. Statistical significance (*** *p* < 0.001) is indicated.

The results of the metabolic activities of mixed biofilms demonstrated the efficiency of all three-culture media in most of the evaluated conditions (Figure 5 and Figure 6). Regardless of the time of mixed biofilm formation (0 h, 4 h, 24 h, and 48 h), the cells remained viable, with high absorbance levels. When *S. aureus* was added to the *T. rubrum* biofilm after 24 h, the metabolic activity was significantly lower than at 0 h, 4 h (*p* < 0.001), and 48 h (*p* < 0.05) (Figure 5A). The BHI medium showed higher metabolic activity, both in biofilms composed of *T. rubrum* + *S. aureus* (Figure 5B) and those of *T. rubrum* + *S. epidermidis* (Figure 6B). The time of bacteria addition influenced biofilm formation between the *T. rubrum* + *S. aureus* in RPMI-1640 (Figure 5A) and the *T. rubrum* + *S. epidermidis* in MHB (Figure 6C). Simultaneous addition of bacterial and fungal inocula (0 h) of *T. rubrum* + *S. epidermidis* in MHB medium resulted in lower metabolic activity compared to the other conditions at 4 h, 24 h, and 48 h (*p* < 0.001) (Figure 6C).

### 3.3. Quantification of Biofilms via the Total Plate Count Method

**Figure 7:**
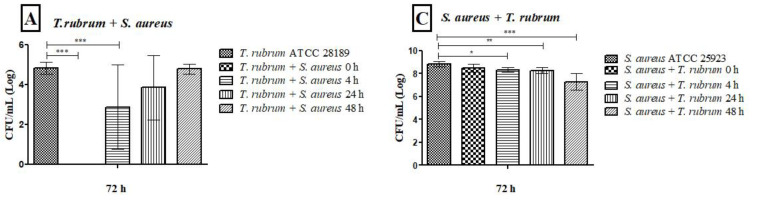

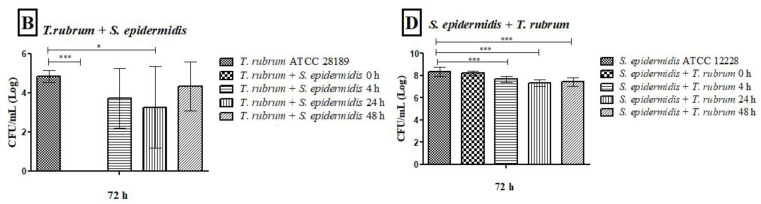
Colony-forming units (CFUs/mL) obtained from monospecies and mixed biofilms of *T. rubrum* (**A**,**B**), *S. aureus* (**C**), and *S. epidermidis* (**D**), formed in Mycosel agar and Mannitol salt agar in the different proposed conditions (0 h, 4 h, 24 h, and 48 h). All biofilms had a total incubation time of 72 h. Statistical significance levels (* *p* < 0.05, ** *p* < 0.01, and *** *p* < 0.001) are indicated.

Our previous assay results showed that the BHI medium effectively grew and maintained monospecies and mixed biofilms for 72 h. Therefore, this medium was chosen for CFU quantification and biofilm image assays. The *T. rubrum* colony counts in the monospecies biofilm, after 48 h, were comparable to those found in the mixed biofilms. However, when bacteria were introduced at earlier time points (0 h, 4 h, and 24 h), the counts were significantly lower than those observed in the monospecies biofilm, especially at 0 h (*p* < 0.001) (Figure 7A,B). The number of bacterial colonies in monospecies biofilms was like that found in mixed biofilms at 0 h. However, at subsequent times (4, 24, and 48 h), bacterial counts were significantly reduced in the mixed biofilms (*p* < 0.05). The results were more pronounced when the bacteria were added after 48 h of *T. rubrum* biofilm formation, indicating that the presence of the dermatophyte hindered bacterial growth (Figure 7C,D). In general, the late addition of bacteria to the mixed biofilm favored fungal growth, while the addition of bacteria in the early stages of mixed biofilm formation favored bacterial growth.

### 3.4. Analysis of Biofilms with Scanning Electron Microscopy (SEM)

**Figure 8:**
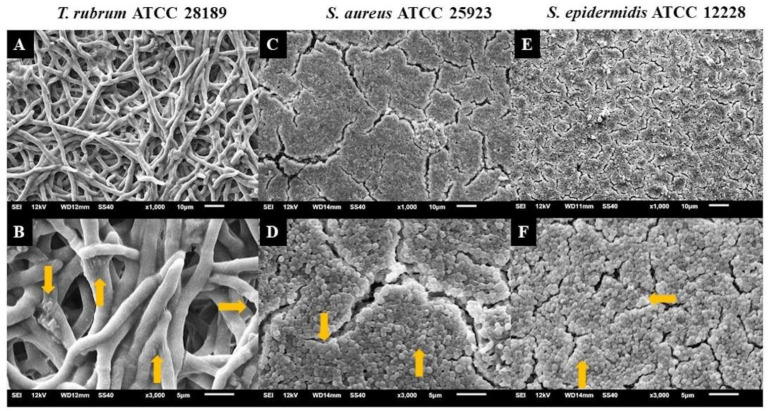
Electron micrographs of biofilms of *T. rubrum* ATCC 28189 (**A**,**B**), *S. aureus* ATCC 25923 (**C**,**D**), and *S. epidermidis* ATCC 12228 (**E**,**F**). All biofilms were analyzed at a total time of 72 h of incubation. Magnifications of 1000× (**upper panels**) and 3000× (**lower panels**). The yellow arrows (**B**,**D**,**F**) point to the polysaccharide material surrounding the hyphae and cocci produced by the dermatophyte and bacteria.

**Figure 9:**
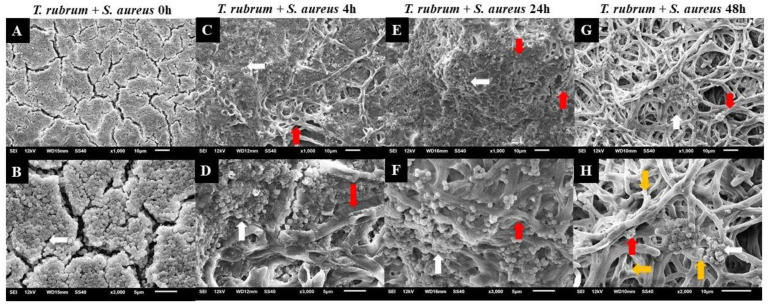
Electron micrographs of biofilms formed by *T. rubrum* ATCC 28189 and *S. aureus* ATCC 25923 when added simultaneously (0 h) (**A**,**B**), with the addition of *S. aureus* suspensions after 4 h (**C**,**D**), after 24 h (**E**,**F**), and after 48 h (**G**,**H**) of *T. rubrum* biofilm formation. All biofilms were analyzed at a total time of 72 h of incubation. The white arrows indicate the structures in the form of cocci of *S. aureus*, the red arrows indicate the hyphae of *T. rubrum*, and the yellow arrows (**H**) point to the polysaccharide material surrounding the hyphae and cocci, produced by the dermatophyte and bacteria. Magnifications of 1000× (**upper panels**) and 3000× (**lower panels**).

**Figure 10:**
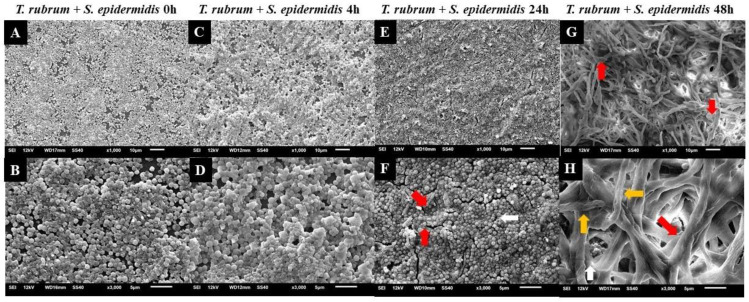
Electron micrographs of biofilms formed by *T. rubrum* ATCC 28,189 and *S. epidermidis* ATCC 12,228 when added simultaneously (0 h) (**A**,**B**), with the addition of *S. epidermidis* suspensions after 4 h (**C**,**D**), 24 h (**E**,**F**), and 48 h (**G**,**H**) of *T. rubrum* biofilm formation. All biofilms were analyzed at a total time of 72 h of incubation. The white arrows indicate the structures in the form of cocci of *S. epidermidis*, the red arrows indicate the hyphae of *T. rubrum*, and the yellow arrows (**H**) point to the polysaccharide material surrounding the hyphae and cocci, produced by the dermatophyte and bacteria. Magnifications of 1000× (**upper panels**) and 3000× (**lower panels**).

Electron micrographs of mixed biofilms established under four different periods were compared to those of monospecies biofilms, using magnifications of 1000× (upper panels) and 3000× (lower panels) (Figure 8, Figure 9 and Figure 10). *T. rubrum* biofilms exhibit a dense network of elongated hyphae extending throughout the biofilm, enveloped by a polysaccharide material indicated by yellow arrows (Figure 8A,B). Biofilms of *S. aureus* (Figure 8C,D) and *S. epidermidis* (Figure 8E,F) present a compact and well-organized mass of rounded bacterial cells surrounded by a polymeric matrix that facilitates cell interaction (yellow arrows).

The presence of hyphae was not observed, while there was a significant abundance of cocci (indicated by white arrows) when *T. rubrum* + *S. aureus* were added simultaneously (Figure 9A,B). These data suggest that the absence of pre-adhesion of *T. rubrum* hampers its development, leading to the predominance of *S. aureus*, corroborating with CFU results. However, when *S. aureus* was added after 4 h of incubation of the dermatophyte (Figure 9C,D), some regions in the electron micrographs revealed the presence of elongated hyphae (red arrows) with numerous overlapping cocci (white arrows). The presence of hyphae was accentuated in biofilm-added bacteria at 24 h (Figure 9E,F) and predominantly at 48 h (Figure 9G,H). Concerning *S. aureus*, a substantial reduction was noted at 24 h and, mainly, at 48 h. Notably, some bacterial cells adhered to the hyphae of the dermatophyte, using them as scaffolds for growth. At 48 h (Figure 9G,H), the presence of hyphae was more evident, and they were covered by a dense matrix that gave the hyphae and cocci a wrinkled appearance (yellow arrows).

Similar outcomes were observed in the mixed biofilm of *T. rubrum* and *S. epidermidis*, as shown in Figure 10. When the bacterial suspension was added simultaneously with *T. rubrum* (Figure 10A,B), a significant number of adhered and well-organized bacterial cells (indicated by white arrows) were observed, which once again prevailed over *T. rubrum*. In this instance, hyphae were not detectable. In the images of the mixed biofilms with bacteria added after 4 h, unlike the biofilm with *S. aureus*, the presence of hyphae was not consistently identified (Figure 10C,D). At 24 h (Figure 10E,F), poorly developed and sparse *T. rubrum* hyphae were observed throughout the biofilm topography (indicated by red arrows), and abundant bacterial cells agglomerated with limited polymeric material, unlike what was observed in the biofilm between *T. rubrum* + *S. aureus*. Only a small number of bacterial cells were observed within the image field once the *S. epidermidis* suspension was introduced to the fungal biofilm after 48 h (Figure 10G,H). *T. rubrum* cells were well-developed throughout the biofilm, resembling monospecies biofilms, but the hyphae were smaller and had a fine polysaccharide material (yellow arrows).

### 3.5. Analysis of Biofilms with Confocal Laser Scanning Microscopy (CLSM)

**Figure 11:**
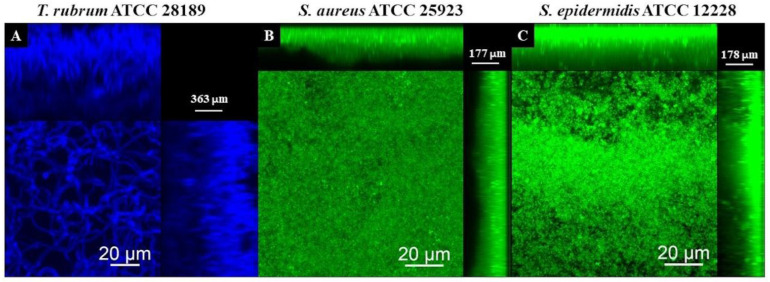
Scanning confocal laser microscopy images of *T. rubrum* ATCC 28189 (**A**), *S. aureus* ATCC 25923 (**B**), and *S. epidermidis* ATCC 12228 (**C**) biofilms. All biofilms were analyzed at a total time of 72 h of incubation. The dermatophyte was stained with Calcofluor White (Blue) and bacteria with Syto 9 (green). All biofilms exhibited dense and cohesive structures. The bacterial biofilms had a thickness of about 177 µm, whereas the *T. rubrum* biofilms were thicker, measuring 363 µm.

**Figure 12:**
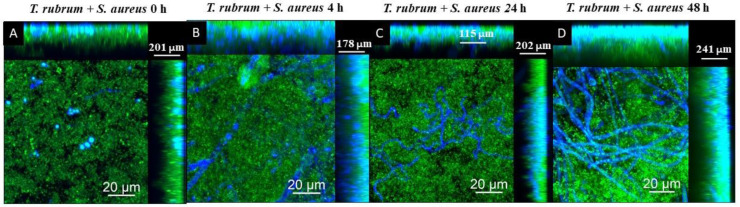
Scanning confocal laser microscopy images of *T. rubrum* + *S. aureus* (**A**–**D**) biofilms under all proposed conditions (0 h, 4 h, 24 h, and 48 h). All biofilms were analyzed at a total time of 72 h of incubation. The biofilms were stained with Calcofluor White (Blue) and Syto 9 (green). In the initial stages of development, *S. aureus* had a notable hindering effect on fungal growth and reduced biofilm thickness. Nevertheless, a significant increase in fungal cells was observed in the later stages, subsequently increasing the biofilms’ overall thickness.

**Figure 13:**
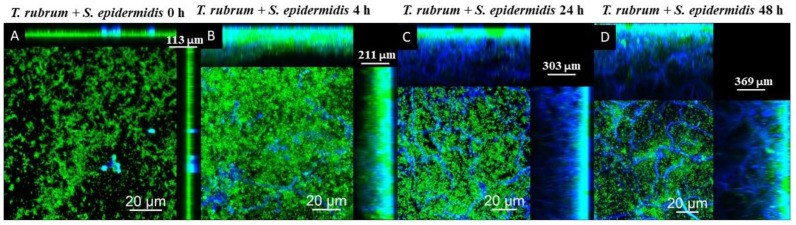
Scanning confocal laser microscopy images of *T. rubrum* + *S. epidermidis* (**A**–**D**) biofilms under all proposed conditions (0 h, 4 h, 24 h, and 48 h). All biofilms were analyzed at a total time of 72 h of incubation. The biofilms were stained with Calcofluor White (Blue) and Syto 9 (green). During the initial phases of development, the growth of fungi was markedly impeded by *S. epidermidis*, leading to a decrease in the thickness of biofilms. However, in the subsequent phases, a noteworthy upsurge in the number of fungal cells was observed, resulting in an overall increase in biofilm thickness.

The CLSM images using Calcofluor White and Syto 9 fluorophores are presented in Figure 11, Figure 12 and Figure 13. The images of the biofilms of the dermatophyte, as well as the bacteria, revealed a dense and cohesive biofilm. In addition, the bacterial biofilms were approximately 177 µm thick (Figure 11B,C), and the *T. rubrum* biofilm was 363 µm thick (Figure 11A). The images of mixed biofilms validate the findings from the SEM observations. In the early stages of formation (Figure 12A and Figure 13A), both bacterial strains strongly inhibited fungal growth and reduced biofilm thickness. Subsequently, a more significant fungal cell was observed at 24 h and 48 h of formation, with considerably developed and elongated hyphae, especially at 48 h; there was an increase in the thickness of these biofilms. Additionally, *Staphylococcus* species were observed among *T. rubrum* hyphae (Figure 11C,D and Figure 12C,D). The thickness of the biofilms at these time points was more remarkable compared to the initial stages.

Correlating all of the performed assays, when microorganisms are added simultaneously, bacterial cell growth is favorable in the biofilm with a scarce presence of fungal cells, which justifies the impossibility of recovering these cells in the CFU assay. The composition of the biofilm in this condition contributed to the presentation of lower biomass and lower thickness. This result suggests that fungal cells provide significant components of the biomass that influence the increase in thickness of the mixed biofilm, especially in the biofilm formed between *T. rubrum* and *S. epidermidis*. Late stage (24 and 48 h) contributes to greater biomass, thickness, and more cells recovered in CFU assays.

## 4. Discussion

Recognizing that nutrient availability significantly influences biofilm growth and architecture [22,25,43,44,45], this research evaluated three different culture media (BHI, MHB, and RPMI-1640) to understand the coexistence/interaction of *T. rubrum*, *S. aureus*, *S. epidermidis* alone or in combination within the biofilm. Although culture media do not provide nutrients that are entirely identical to the components of human bodily fluids, they mimic and create an environment conducive to the growth of a wide range of microorganisms [46].

The results indicate that all media tested can promote the development of all biofilms, although BHI promoted the best growth in metabolic activity and biomass of monospecies and mixed biofilms under all conditions tested. The BHI medium provides a rich source of nutrients that simulate the nourishing environments within the human body. The peptone in BHI is derived from protein hydrolysis, serving as a vital source of amino acids and peptides. In the human body, dietary proteins are broken down into amino acids, fundamental building blocks for various physiological processes, such as tissue repair and immune function. Glucose, another constituent of BHI, mirrors the primary energy source for cells in the human body, ensuring readily available energy for microorganisms in culture. Sodium chloride, also present in BHI, helps maintain osmotic balance, a critical factor for microbial growth. Phosphates, essential components of nucleotides that constitute DNA and RNA, are found in disodium phosphate within BHI, serving as a buffer and providing the necessary phosphate ions for various microbial metabolic processes. In addition, in agreement with previous studies, the BHI medium provides better development of polymicrobial biofilms, whether between fungal species or fungi and bacteria [27,47].

Moreover, the simultaneous introduction of microorganisms resulted in notably lower biomass at 0 h, emphasizing the initial coexistence challenges. However, as time advanced, the biomass significantly increased, indicating a complex interplay between fungal and bacterial species during biofilm formation. A crucial observation from this study is the temporal sensitivity observed in mixed biofilms. Substantial differences in biomass were evident when microorganisms were introduced simultaneously or within the initial hours of biofilm development. This temporal influence underscores the importance of considering the timing of microbial interactions in mixed biofilm research.

Regarding metabolic activity, the quantification of *T. rubrum* biofilms indicates a substantial increase up to 72 h across all tested media. For *S. aureus* and *S. epidermidis* biofilms, BHI and RPMI-1640 are more conducive to higher metabolic activity at 24 h than MHB. Notably, both BHI and MHB maintain consistent metabolic activities from 24 to 72 h, while RPMI-1640 shows a slight decrease in the metabolic activity of bacterial communities. This result emphasizes the medium-dependent nuances in the metabolic profiles of bacterial biofilms. Furthermore, BHI emerges as a favorable medium, promoting higher metabolic activity in mixed biofilms of *T. rubrum* with *S. aureus* or *S. epidermidis.* The temporal aspect of bacteria addition significantly influences mixed biofilm formation, with lower metabolic activity observed when *S. aureus* is added after 24 h in mixed biofilms. Simultaneous addition at 0 h also results in lower activity in *T. rubrum* + *S. epidermidis* biofilms compared to other time points. These findings underscore the intricate temporal dynamics governing the metabolic activities of mixed biofilms and contribute valuable insights into medium-dependent variations in biofilm behavior.

Electron micrographs of mixed biofilms showed a more significant presence of bacterial cells in the initial conditions (0 and 4 h). In the late interaction conditions (24 and 48 h), there was less presence of cocci, with hyphae dominating. This observation is consistent with the literature on biofilms of *S. aureus*, *Escherichia coli*, and *C. albicans* [26,48,49]. In addition, these findings may reveal the importance of adhesion for fungal cells, showing a significant impairment in the growth of *T. rubrum*. Dermatophyte biofilm formation involves a well-coordinated process with distinct stages. Among these, adhesion is a prerequisite for biofilm development and plays a critical role in initiating the colonization of host tissues and the subsequent establishment of infection [50,51,52,53]. During this period, contact between the conidia, hyphal fragments, and substrate is established, accompanied by the secretion of adhesins by the arthroconidia, facilitating attachment to the host stratum corneum [54]. As documented by Zurita and Hay [50], the adhesion of dermatophytes follows a time-dependent pattern, occurring between 3 and 4 h. Another critical factor is the availability of nutrients in biofilms.

In many cases, there is competition between the different microorganisms that make up the polymicrobial biofilm. Since the metabolism of the tested *Staphylococcus* species is more accelerated compared to *T. rubrum*, this may justify the massive growth in bacterial cells when added at 0 and 4 h, inhibiting the uptake of nutrients and the development of *T. rubrum*. Similar behavior has been reported in mixed biofilms containing *Candida* spp. and *T. rubrum* [18].

Observations of biofilms of *Staphylococcus* spp. and *T. rubrum* using CLSM revealed dense biofilms with cells bound together by an extracellular matrix. The fungal biofilm’s thickness was twice as large as that reported for bacterial biofilms. *Staphylococcus* species biofilms require the synthesis of extracellular matrix components for cellular maturation and the formation of complex three-dimensional structures. This process is mediated by cell–cell adhesion and matrix components, resulting in critical tower-like structures for pathogenesis [55,56]. Dermatophytes have been reported to form dense biofilms similar in thickness to those observed in this study [19].

From a clinical standpoint, the emergence of mixed biofilms containing *Staphylococcus* spp. and *T. rubrum* poses challenges in diagnosing, treating, and managing patients. The temporal changes observed in this study, specifically in the population dynamics within biofilms, offer valuable insights for clinical decision-making during treatment. Prior studies have indicated that in the biofilm environment, microorganisms can secrete bioactive compounds with the potential to induce greater resistance or alterations in virulence [48,49,50,51,52,53,54,55,56,57]. Furthermore, bridging the gap between laboratory studies and practical applications is essential for translating scientific insights into effective clinical practices. Incorporating in vivo models could enhance the relevance of our findings, thus bringing them closer to real-world clinical scenarios. Addressing these aspects could contribute to a deeper comprehension of the underlying mechanisms and provide more comprehensive insights for improving clinical outcomes.

## 5. Conclusions

Our study provides new insights into in vitro biofilm formation involving *Staphylococcus* spp. and *T. rubrum*, revealing variable characteristics influenced by interaction conditions and nutrient availability. Biofilms formed in BHI medium exhibit notable enrichment in biomass, metabolic activity, and thickness. Notably, the initial stages (0 h, 4 h, and 24 h) show bacterial dominance, shifting to *T. rubrum* predominance at 48 h. This research enhances our comprehension of mixed biofilm interactions, emphasizing their complexity and revealing new opportunities for investigating microorganism interactions in polymicrobial biofilms and comprehending host interactions, pathogenesis, and potential therapeutic strategies.

## Data Availability

The original contributions presented in the study are included in the article. Further inquiries can be directed to the corresponding author.
